# Post-transcriptional control of the mammalian circadian clock: implications for health and disease

**DOI:** 10.1007/s00424-016-1820-y

**Published:** 2016-04-23

**Authors:** Marco Preußner, Florian Heyd

**Affiliations:** Institute of Chemistry and Biochemistry, Laboratory of RNA Biochemistry, Free University Berlin, Takustrasse 6, 14195 Berlin, Germany

**Keywords:** Circadian clock, Post-transcriptional regulation of gene expression, Chronomedicine, Alternative splicing, mRNA stability, Translation

## Abstract

Many aspects of human physiology and behavior display rhythmicity with a period of approximately 24 h. Rhythmic changes are controlled by an endogenous time keeper, the circadian clock, and include sleep-wake cycles, physical and mental performance capability, blood pressure, and body temperature. Consequently, many diseases, such as metabolic, sleep, autoimmune and mental disorders and cancer, are connected to the circadian rhythm. The development of therapies that take circadian biology into account is thus a promising strategy to improve treatments of diverse disorders, ranging from allergic syndromes to cancer. Circadian alteration of body functions and behavior are, at the molecular level, controlled and mediated by widespread changes in gene expression that happen in anticipation of predictably changing requirements during the day. At the core of the molecular clockwork is a well-studied transcription-translation negative feedback loop. However, evidence is emerging that additional post-transcriptional, RNA-based mechanisms are required to maintain proper clock function. Here, we will discuss recent work implicating regulated mRNA stability, translation and alternative splicing in the control of the mammalian circadian clock, and its role in health and disease.

## Basic principles of circadian biology

**Fig. 1:**
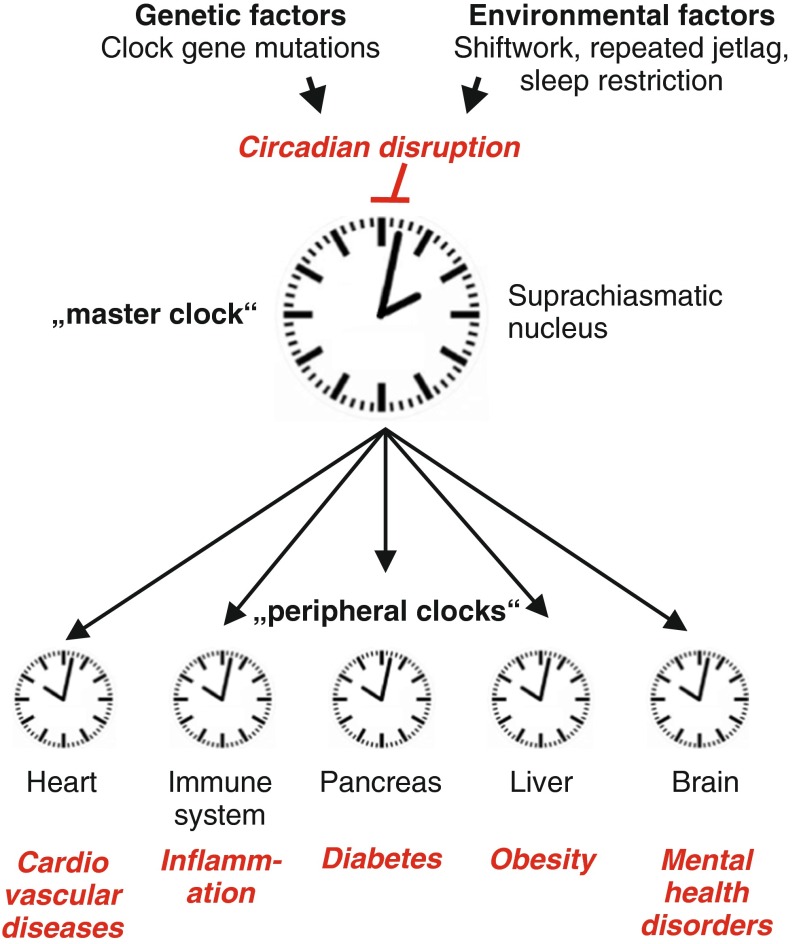
Hierarchical organization of the circadian clock. The master pacemaker of the body is located in the suprachiasmatic nucleus (SCN) which receives direct light input through the eye. Signals from the SCN then synchronize local clocks in the peripheral organs to one another and to the environment. Therefore, peripheral clocks show about 4-h phase delay with respect to the SCN [45]. Malfunctioning of the circadian clock (*top*) is associated with diverse diseases in the peripheral organs (*bottom*)

The daily cycle of night and day, leading to oscillating changes in light-dark and temperature conditions, influences all aspects of life. This is true for animals in the wild but also for human beings, who, despite being independent of the sun for light and heat, still follow a 24-h rhythm. The predictable daily repetition of sleep-wake cycles, activity and rest phases, food intake and digestion, etc. allows the body to anticipate requirements and prepare for upcoming tasks. Therefore, many physiological and behavioral aspects are controlled in a periodic, daily manner. For example, blood pressure and digestive activity increase in the morning whereas the production of melatonin is increased in the evening. This occurs in anticipation of activity and food intake or sleep, respectively. The internal system that produces such a 24-h rhythm is called the circadian clock, a cell autonomous oscillator with a period of around 24 h that is present in nearly every cell of the body. In mammals, the tight connection and crosstalk between individual cells of the same tissue lead to the formation of interconnected, organ-specific clocks. This ensures that cells of the same organ are in the same phase and in addition, the oscillation of a (large) group of connected cells is more robust than the oscillation of a single cell alone [21]. The whole system is controlled by, synchronized and aligned with the environment through signals emanating from the suprachiasmatic nucleus (SCN), a region in the brain referred to as the central or master clock [41]. The SCN itself receives direct light input from the eye, which is the most important external cue to synchronize the circadian clock [13]. In the SCN, light activates signaling cascades resulting in neuronal or humoral output, which transmit the signal to other cells and organs collectively called peripheral clocks (Fig. 1). The exposure to light thus acts as a daily resetting signal for the SCN, which then synchronizes peripheral clocks accordingly. In changing external conditions, e.g., upon traveling to another time zone, the body can readjust to the new light-dark phase, which then allows an adaptation of physiological functions to the new environment. As the circadian clock is relatively stable against altered extracellular conditions, this adjustment requires approximately 1 day per hour of phase shift. Importantly, physiological and behavioral cycles with a period of approximately 24 h are maintained in the absence of external cues showing that the circadian clock is a self-sustained system producing a stable oscillation. The features described above, a period of approximately 24 h, entrainability to changing external conditions and the persistence in the absence of external cues are three criteria that need to be fulfilled for an event to be circadian. A fourth criterion is temperature compensation, meaning that a circadian event is stable over a wide temperature range.

**Fig. 2:**
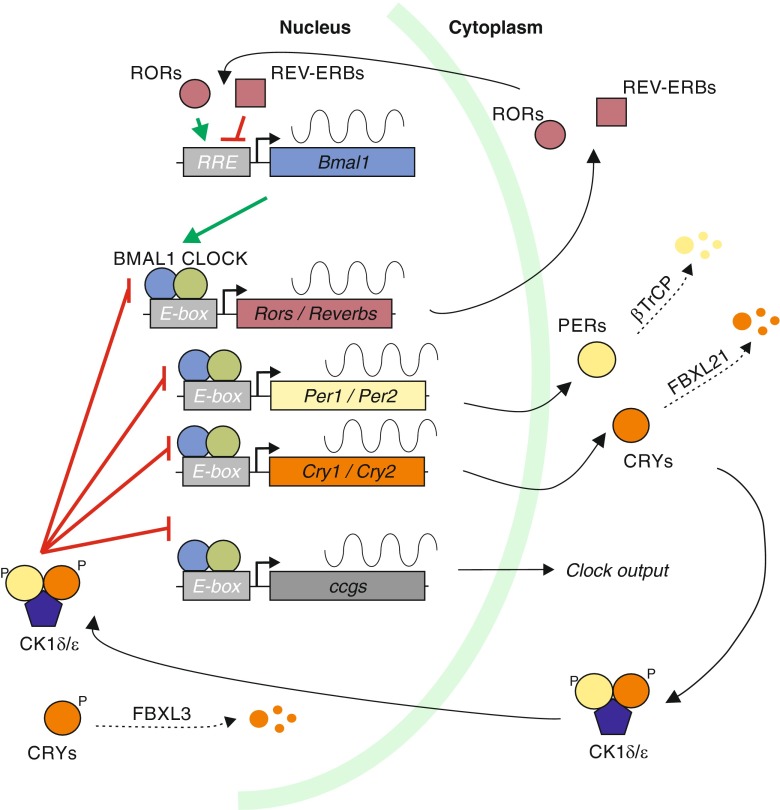
The mammalian circadian clock consists of negative and positive transcriptional feedback loops. At the core of the circadian clock, the BMAL1/CLOCK heterodimer drives oscillating expression of clock-controlled genes (ccgs) with E-box containing promoters. Another layer of regulation is achieved through a second group of ccgs—RORS and REV-ERBs—that regulate BMAL1 transcription. The clock output, such as altered metabolism, is achieved through many genes with E-box containing promoters collectively shown as ccgs (*gray*). See main text for further details and abbreviations

Circadian clocks are not restricted to mammals or eukaryotes; instead, corresponding mechanisms, some depending on the redox state and not on a transcription-translation feedback loop (Fig. 2 and see below), are present in all three domains of life [14]. This suggests that the presence of a circadian clock, allowing the anticipation of daily rhythms, confers an evolutionary advantage to unicellular as well as multicellular organisms that justifies the production and maintenance of an elaborate clockwork.

## The circadian clock in health and disease

In humans, many fundamental parameters of the body such as heart rate, blood pressure, body temperature, hormone and cytokine secretion, metabolism, and cerebral activity, show a circadian profile, pointing to a strong influence of the circadian clock on health and disease [62]. The connection and its importance become already apparent when traveling to a different time zone. This leads to a misalignment of the circadian clock with the environment and can cause discomfort and drowsiness, the typical jet lag syndromes. Similarly, “social jet lag”, e.g. the sudden change of daily rhythms at weekends, can disturb the circadian clock and may contribute to the “Monday morning blues” [58]. These examples show that already a temporary disturbance or misalignment of the circadian clock with the environment is sufficient to cause physical and mental uneasiness. In an experimental setting, chronic jet lag increased the mortality rate of old mice [11], further underlining the connection between the circadian system and well-being. Not surprisingly, continued disruption of the circadian rhythm either by genetic or by environmental factors is associated with a variety of diseases in humans (Fig. 1). Especially prominent are cardiovascular diseases, inflammation, cancer, mental health and metabolic disorders, such as diabetes or obesity. The range of diseases may partially reflect the prominent role of the circadian clock in controlling the cell cycle and metabolism [3]. The connection between the circadian clock and pathological conditions has been made and confirmed in different settings such as model organisms, human genetics, and their increased occurrence in shift workers. As an example, long-term shift work has been suggested to increase the risk for colorectal, prostate, and breast cancer as well as cardiovascular and metabolic disorders [57]. Such connections were also found in mice with a genetically disrupted or disturbed clockwork, as these mice show increased incidence of cancer, insulin resistance, and obesity for example [53]. The latter connection is further underlined by the correlation of the time of food intake with weight gain and the development of obesity [2].

The manifestation of several other diseases does not show a direct connection with the circadian system but the severity of their symptoms follows a circadian pattern. Examples include asthma, allergies, or epileptic seizures that preferentially happen at particular times of the day [35, 43], probably associated with circadian histamine and cortisol levels in the blood [7]. Consequently, current efforts are directed toward integrating circadian biology into treatment schedules. If symptoms follow a circadian rhythm, the treatment schedule could be adjusted accordingly; in addition, the body may show very different reactions to the same dose of a drug administered in the morning or the afternoon [6]. First studies have included this aspect in treatment regimens and promising results have been obtained, e.g., in a low-dose prednisone chronotherapy in rheumatoid arthritis patients [8]. Another very active field of research is cancer chronotherapy, as the efficacy as well as the tolerability of chemotherapy shows a circadian profile [44]. While these approaches have a huge potential to improve therapies when applied in general, the future goal of chronomedicine is a personalized treatment schedule based on individual circadian parameters. This will further increase the effectiveness of chronomedicine, as individuals have different chronotypes and the phase and amplitude of the circadian clock changes with aging [50].

In addition to the examples described above, the circadian clock is also connected to neurological and mental disorders. Circadian rhythms play an obvious role in controlling sleep cycles and have been linked to seasonal affective disorder (SAD), a mood disorder with symptoms of depression only during a certain time of the year, depression and neurodegenerative diseases [1, 55, 56]. Another prominent example is the familial advanced sleep phase syndrome, an inheritable disease caused by a point mutation in the PER2 gene. The PER2 S662G mutation destroys a phosphorylation site and thereby changes the stability of the protein, resulting in an altered period and disturbed sleep-wake cycles in the affected individuals [54].

Together, these examples show the strong influence of circadian biology on health and diseases. An extreme example to further illustrate the connection is the dramatically altered mortality of mice when challenged with the same dose of LPS at different times of the day [18], which may be partly explained by micro RNA (miRNA)-based regulation of BMAL1 (see below). In humans, even birth and death show higher occurrence at particular circadian times, with more babies being born in early morning hours [25] and fatalities caused by cardiovascular events peaking between morning and noon [42], emphasizing the central role of the circadian clock in fundamental aspects of life.

## Molecular principles of the circadian clock

Rhythmic changes in behavior and physiology are controlled and mediated by extensive circadian changes in gene expression. At the center of the mammalian molecular clock (Fig. 2) is the heterodimeric transcription factor CLOCK/BMAL1 that binds to E-Box containing promoters to activate transcription of up to 10 % of expressed genes (clock-controlled genes, ccgs; [49]). Among the ccgs are the period (PER) and cryptochrome (CRY) genes, that, upon transcription and translation, accumulate in the cytoplasm. Here, the stability of PER and CRY proteins is regulated by ubiquitination—mediated by the E3-ubiqutin ligases βTRCP1 and FBXL21, respectively—and proteasomal degradation [20, 48, 51, 61]. PER/CRY dimers become phosphorylated by the CK1δ/ε kinase and translocate into the nucleus when sufficient amounts of protein have accumulated. In the nucleus, PER/CRY heterodimers inhibit transcription through CLOCK/BMAL1 thus shutting down the expression of ccgs, including themselves; this starts a new cycle once the PER/CRY proteins are degraded and CLOCK/BMAL1 activity is restored [29]. In the nucleus, the stability of PER and CRY proteins is also regulated by ubiquitination. Here, the E3-ubiqutin ligases FBXL3 promotes ubiquitination and proteasomal degradation of CRYs [51]; this activity is antagonized by FBXL21 [20, 61]. A further feedback mechanism is provided through another family of ccgs, RORs and REV-ERBs, which control the transcription of BMAL1 [17]. RORs and REV-ERBs are nuclear receptors that bind to a so-called REV-ERBs/ROR-binding element (RRE) in the promoter region of BMAL1 and regulate its transcription antagonistically. Together, a negative feedback loop is established, which operates with a period of approximately 24 h, and forms the basis for autonomous clocks that are found in a wide variety of cell types in mammalian organisms. In addition to this well-studied transcription-translation feedback loop, post-transcriptional mechanisms have gained increasing attention in the last years, as they were shown to be indispensable for the regulation of the circadian clock. Here, we will focus on recently described post-transcriptional, RNA-based mechanisms in mammalian systems and their impact on circadian biology. For posttranslational modifications and their central role in controlling circadian rhythms, we refer to several recent reviews [29, 39, 52] and references therein.

## Emerging RNA-based post-transcriptional mechanisms controlling the mammalian circadian clock

Until recently, E-Box-mediated transcription and its inhibition in a negative transcription-translation feedback loop were believed to be the main rhythmicity inducing mechanism. However, results obtained in the last years indicate that less than 30 % of circadian messenger RNAs (mRNAs) are regulated by de novo transcription, suggesting that other mechanisms account for the majority of circadian gene expression [30, 40]. Indeed, several recent reports confirmed that many, if not all, steps in the life cycle of a (pre-) mRNA can be regulated in a circadian manner thus contributing to circadian gene expression. Core nuclear processing events of mammalian pre-mRNAs are capping, splicing, polyadenylation, and export to the cytoplasm, which will be discussed in the context of circadian biology in the following.

**Fig. 3:**
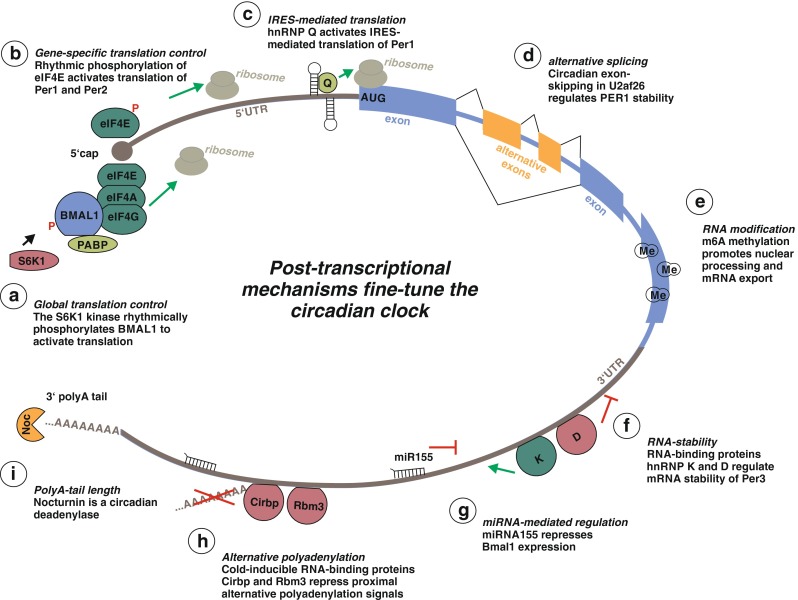
Post-transcriptional mechanisms acting on the (pre-) mRNA to control the mammalian circadian clock. Before an mRNA becomes translated, several highly regulated processing steps have to occur. This includes capping at the 5’ end, splicing, and polyadenylation at the 3’ end. Furthermore, translation and degradation of the mature mRNA are regulated processes as well. The figure summarizes post-transcriptional events in the life cycle of a (pre-) mRNA that have been reported to directly influence the circadian clock and/or to be controlled in a circadian manner. See main text for further details

Processing of mammalian pre-mRNA starts with the modification of its 5’ end, the formation of the cap-structure. Regulation of capping has not been documented to be under circadian control, but this may simply reflect that is was not systematically analyzed in circadian settings. It was indeed suggested, that knock down of the RNMT methylase required for cap formation prolongs the circadian period [15], which could indicate that cap formation and its effect on mRNA stability or translation are involved in controlling the circadian cycle. Consistent with this idea, two recent reports have documented that translation through the cap-binding protein eIF4E is under circadian, phosphorylation-dependent control (Fig. 3a, b). Upon rhythmic phosphorylation by the mTOR-effector kinase, ribosomal S6 protein kinase 1 (S6K1), the core circadian transcription factor BMAL1 associates with the translation machinery in the cytoplasm and globally promotes protein synthesis. Phosphorylated BMAL1 interacts with the translation machinery through the cap-binding protein complex eIF4E/A/G and the polyA-binding protein thus controlling cap-dependent translation in a circadian manner [34]. The precise role of BMAL1 in this regulation remains unknown, but a model is conceivable, in which phosphorylated BMAL1 provides a scaffold for more efficient assembly of the translational machinery including polyA-binding protein to generally increase cap-dependent translation. It is interesting to note that PolII-mediated transcription has also been proposed to be globally controlled in a circadian manner. RNA-sequencing of liver mRNAs revealed a peak of intron-containing pre-mRNAs at ∼CT15 (circadian time), which might be explained by a general activation of transcription shortly before [30]. Together, these data suggest that in addition to the specific regulation of individual genes, several steps in global gene expression are under control of the circadian clock.

Translation efficiency can also be directly modulated through phosphorylation of the eIF4E protein [9]. Here, a light- and circadian clock-regulated MAPK/MNK pathway phosphorylates eIF4E to specifically enhance translation of PER1 and PER2 mRNAs. This effect was specific for PER1 and PER2 mRNAs, likely depending on the sequence of the respective 5’ untranslated regions (UTRs), but the nature of the required *cis*-acting element and potential coregulated mRNAs have not been defined. The widespread use of regulated translation to generate circadian gene expression was further confirmed in two recent large-scale ribosome-profiling approaches. These studies identified cycling transcripts that are additionally controlled at the level of translation but also found hundreds of non-cycling mRNAs displaying rhythmic ribosome footprints [23, 24]. These results suggest a prominent role for regulated efficiency of translation to control circadian gene expression. One example for RNA-based translational control are short upstream open reading frames (uORFs) that can repress translation of the primary ORF [38]. Such uORFs were identified in core clock components such as BMAL1 and CLOCK, which may contribute to the regulation of the circadian clock. Whether these particular uORFs are functionally important remains to be seen; the finding that knock down of a protein required for reinitiation of translation downstream of uORFs leads to a shortened period provides evidence that at least some uORFs, whose precise nature remains to be identified, are involved in circadian control [24].

Translation of PER1 is further modulated by an internal ribosomal entry site (IRES) in the 5’ UTR of its mRNA (Fig. 3c). IRES-mediated translation allows direct recruitment of the 40S ribosome subunit to the vicinity of the initiation codon and is therefore independent of the classical cap-dependent translation machinery. The IRES element of PER1 was sufficient for rhythmic protein expression of a bicistronic luciferase reporter in synchronized cells thus providing another means to control PER1 expression post-transcriptionally. IRES-mediated translation required rhythmic interaction of the PER1 IRES element with the heterogeneous nuclear ribonucleoprotein Q (hnRNP Q), which activates non-canonical PER1 translation in a circadian manner [33]. The importance of IRES-mediated translation was further confirmed by mathematical modeling and might be regulated through rhythmic phosphorylation of hnRNP Q [32]. Similarly, hnRNP Q and polypyrimidine tract-binding protein (PTB) have been shown to modulate IRES-mediated translation of Reverbα [28].

Another major RNA-processing event, alternative splicing, has been found to be regulated in a circadian manner and to control different aspects of the circadian clock. In general, alternative splicing is a widespread mechanism that can control gene expression in response to changing extracellular conditions [19]. Systematic evidence for mammalian circadian alternative splicing came only recently from a microarray-based approach that suggested many exons to be alternatively spliced in a circadian manner in mouse liver. Furthermore, this microarray suggested circadian mRNA expression of 62 RNA-binding proteins, which might be involved in the regulation of circadian RNA-processing events [37]. The first functional connection between alternative splicing and the mammalian circadian clock was described for the U2AF26 gene that shows circadian and light-induced skipping of exons 6 and 7 in mouse cerebellum (Fig. 3d). This exon-skipping event does not result in a shorter protein product, but, through inducing a frameshift, allows translation into the supposed 3’ UTR adding a new C-terminus to the protein. Interestingly, this new domain shows homology to parts of a drosophila protein called Timeless, which is a central component of the fly circadian clock. This novel U2AF26 isoform localizes to the cytoplasm and, in analogy to Timeless, interacts with PER1 to control its stability. Furthermore, U2AF26-deficient mice show defects in the core molecular clockwork and clock resetting under jet lag conditions, providing evidence for a functional role of U2AF26 alternative splicing in controlling the circadian clock [47]. Another functionally interesting case of alternative isoform expression has been described for melanopsin (Opn4). Here, different isoforms generated by coupled alternative splicing and polyadenylation mediate different responses to light [22]; the regulation of these alternative splicing events and a potential rhythmic production of these isoforms are interesting question to be addressed in future work. Two of the core components of the mammalian circadian clock, CRY1 and PER2, have also been suggested to be alternatively spliced in a circadian manner [4, 46]. However, whether these splicing events are functionally and physiologically relevant remains to be shown.

Further processing events of an mRNA are the addition of a polyA tail, mRNA export into the cytoplasm and the control of mRNA stability, frequently regulated through sequences located in the 3’ UTR. The general efficiency of nuclear mRNA processing and export has been reported to be enhanced through m6A methylation, both in a global manner and specifically for certain core clock transcripts (Fig. 3e; [15]). Consistent with a role of m6A methylation in the circadian cycle and mRNA export, knock down of the methylase Mettl3 elongated the circadian period and led to a general defect in nuclear export of mRNA. While knock down studies and pharmacological inhibition of RNA methylation provide evidence for this regulation, it remains to be shown whether Mettl3 activity and RNA m6A methylation patterns are indeed controlled in a circadian manner.

The 3’ UTR contains many regulatory regions that influence gene expression post-transcriptionally, with important implications for circadian biology. An example are RNA-binding proteins that have been implicated in the control of the circadian clock through controlling mRNA stability of clock components. The hnRNPs K and D both interact with the 3’ UTR of PER3 and antagonistically affect its mRNA stability (Fig. 3f). While hnRNP K stabilizes PER3 mRNA, hnRNP D destabilizes and decreases the amplitude of PER3 mRNA expression [26]. In a similar manner, oscillating cytoplasmatic levels of hnRNP D have been implicated in the modulation of CRY1 mRNA stability [59]. While rhythmic protein expression has been shown or suggested for some hnRNPs such as hnRNP Q [27], hnRNP D [59] and a U2AF26 variant (see above, [47]) this remains to be shown for other RNA-binding proteins. Given the large percentage of circadian mRNAs that are not regulated by de novo transcription, these cases of regulated mRNA stability may only be examples of a widely used mechanism. RNA-binding proteins could selectively stabilize/destabilize target mRNAs at a certain time of the day, thereby yielding a circadian expression profile without changing transcription rates. While the presence of such a regulatory mechanism appears likely, a systematic evaluation of circadian mRNA stability has yet to be performed.

A similarly but equally speculative mechanism would be the presence of circadian miRNAs that could regulate stability or translation of several or many target mRNAs. As a proof of principle it has been shown that miRNA155 can directly target the 3’ UTR of the BMAL1 mRNA to suppress its expression (Fig. 3g). In this case, expression of miRNA155 and its control of BMAL1 expression has been suggested to regulate the different outcomes of the macrophage-mediated innate immunity when stimulated with LPS at different times of the day. Upon macrophage-specific deletion of BMAL1, mice loose the dependence of the mortality rate on the time of the day upon LPS challenge, providing evidence for the involvement of BMAL1 in this phenomenon [10]; however, to confirm a role of miRNA155 in this pathway, such experiments will have to be repeated in miRNA155 deficient animals.

The length of the 3’ UTR can be modulated through alternative polyadenylation, which has recently been linked to the circadian clock. Two cold-inducible RNA-binding proteins have been reported to regulate the choice of alternative polyadenylation sites in the 3’ UTR of clock-controlled genes and may be involved in synchronization of peripheral clocks in response to changing body temperature [36]. Upregulation of Cirbp and Rbm3, as observed under cold-shock, repressed the usage of proximal polyadenylation signals, resulting in prolonged 3’ UTRs of interacting target genes (Fig. 3h). Extended 3’ UTRs may harbor more miRNA-binding sites than the shorter versions [12], which could be associated with a decrease in translation, but further work is needed to confirm such a model in circadian settings. mRNA stability and translation efficiency are furthermore regulated by the length of the polyA tail. Deadenylases are believed to specifically deadenylate certain target mRNAs to yield a selective destabilization. The deadenylase Nocturnin (Noc) is expressed in a circadian manner (Fig. 3i; [5]) and is thus an interesting target to follow this connection. Although Nocturnin knockout mice show resistance to diet-induced obesity and show altered polyA length in a variety of mRNAs [16], these transcripts did not display a circadian change in abundance or polyA length [31]. The potential role of Nocturnin in circadian gene expression and metabolism thus remains to be clarified.

In summary, increasing evidence supports a strong connection of post-transcriptional regulatory events with the mammalian circadian clock. However, investigating the full impact of individual mechanisms such as regulated alternative splicing, mRNA stability or polyadenylation on circadian biology remains a challenging task for the future. In all these cases, global analysis, mechanistic details, and the functional impact of individual components of a group of coregulated (pre-) mRNAs promise exciting discoveries in the coming years.

## Conclusions and perspectives

The circadian clock impacts on diverse aspects of human physiology, as has been well-established through elaborate research in the past. Disruption of core clock gene oscillation, either through genetic mutations within these genes or through environmental conditions that interfere with the circadian rhythm, is associated with a variety of diseases. Consequently, chronomedicine is increasingly acknowledged to have a huge potential in improving treatment regimens. Although therapies based on individual chronotypes are still some way in the future, the insights gained through basic research will finally enable the implementation of personalized therapy schedules.

A well-characterized inheritable disease affecting the circadian clock is familial advanced sleep phase syndrome, which is associated with mutations in the CK1δ gene or a point mutation in the PER2 gene which prevents CK1-mediated phosphorylation [54, 60]. This is one example highlighting the importance of posttranslational modifications of core clock genes for circadian biology. In fact, work from the last years conclusively showed that in addition to the core transcription-translational feedback loop, phosphorylation, ubiquitination, and controlled protein degradation are indispensable for proper clock function [29, 39, 52]. Interestingly, circadian control has been investigated mainly at the transcriptional or the posttranslational level, and RNA-based mechanisms contributing to this regulation are only beginning to emerge. Therefore, the contribution of regulated mRNA processing remains exemplary, and a global impact on circadian biology remains to be confirmed. However, given the finding that the majority of circadian mRNAs is not regulated by de novo transcription [30, 40] and the evidence from the studies presented here, we predict that some, if not all, of the mechanisms discussed above will be shown to play prominent and widespread roles in circadian biology. We also consider it likely that after the identification of trans-acting factors involved in RNA-based control of the circadian clock, connections to diseases will follow. A better understanding of the molecular mechanisms that govern the mammalian circadian clock is thus required to first identify such connections, which may then present opportunities for new therapeutic concepts.
